# Associations of Fine Particulate Matter Species with Mortality in the United States: A Multicity Time-Series Analysis

**DOI:** 10.1289/ehp.1307568

**Published:** 2014-05-06

**Authors:** Lingzhen Dai, Antonella Zanobetti, Petros Koutrakis, Joel D. Schwartz

**Affiliations:** Department of Environmental Health, Harvard School of Public Health, Boston, Massachusetts, USA

## Abstract

Background: Epidemiological studies have examined the association between PM_2.5_ and mortality, but uncertainty remains about the seasonal variations in PM_2.5_-related effects and the relative importance of species.

Objectives: We estimated the effects of PM_2.5_ species on mortality and how infiltration rates may modify the association.

Methods: Using city–season specific Poisson regression, we estimated PM_2.5_ effects on approximately 4.5 million deaths for all causes, cardiovascular disease (CVD), myocardial infarction (MI), stroke, and respiratory diseases in 75 U.S. cities for 2000–2006. We added interaction terms between PM_2.5_ and monthly average species-to-PM_2.5_ proportions of individual species to determine the relative toxicity of each species. We combined results across cities using multivariate meta-regression, and controlled for infiltration.

Results: We estimated a 1.18% (95% CI: 0.93, 1.44%) increase in all-cause mortality, a 1.03% (95% CI: 0.65, 1.41%) increase in CVD, a 1.22% (95% CI: 0.62, 1.82%) increase in MI, a 1.76% (95% CI: 1.01, 2.52%) increase in stroke, and a 1.71% (95% CI: 1.06, 2.35%) increase in respiratory deaths in association with a 10-μg/m^3^ increase in 2-day averaged PM_2.5_ concentration. The associations were largest in the spring. Silicon, calcium, and sulfur were associated with more all-cause mortality, whereas sulfur was related to more respiratory deaths. County-level smoking and alcohol were associated with larger estimated PM_2.5_ effects.

Conclusions: Our study showed an increased risk of mortality associated with PM_2.5_, which varied with seasons and species. The results suggest that mass alone might not be sufficient to evaluate the health effects of particles.

Citation: Dai L, Zanobetti A, Koutrakis P, Schwartz JD. 2014. Associations of fine particulate matter species with mortality in the United States: a multicity time-series analysis. Environ Health Perspect 122:837–842; http://dx.doi.org/10.1289/ehp.1307568

## Introduction

Over the past few decades, there has been much research on the adverse effects of ambient particulate matter (PM). A number of studies have used fine PM (PM_2.5_; particles ≤ 2.5 μm in aerodynamic diameter) as an exposure metric and estimated the effects of PM_2.5_ on human health (Laden et al. 2006; Ostro et al. 2006; Pope and Dockery 2006; Zanobetti and Schwartz 2009). Meanwhile, researchers have found that some PM_2.5_ species significantly modify PM_2.5_-related effects (Franklin et al. 2008; Lippmann et al. 2006; Zanobetti et al. 2009). PM_2.5_ consists of many chemical components that originate from various sources, such as traffic, biomass burning, and coal combustion. The U.S. National Research Council has emphasized the importance of examining the risk of PM species (National Research Council 2004). Determining the differential toxicity of PM_2.5_ species and identifying species with greatest toxicity is of great importance to emission-control strategies and regulations.

The U.S. Environmental Protection Agency (EPA) established the PM_2.5_ Speciation Trends Network (http://www.epa.gov/ttnamti1/speciepg.html) in 2000. Speciation sampling was conducted every third or sixth day, which limits statistical power for analysis of responses to acute exposure and also prevents the examination of, for example, 2-day moving averages of exposure, which most studies find more strongly associated with mortality and hospital admissions than single-day exposures. As a result, a limited number of studies have investigated the toxicity of PM_2.5_ components. These investigations have reported numerous components that may be responsible for particle toxicity, such as elemental and organic carbon, sulfate, nitrate, and metals including zinc, nickel, iron, potassium, and chromium (Atkinson et al. 2010; Bell et al. 2009; Franklin et al. 2008; Ostro et al. 2006; Valdes et al. 2012; Zhou et al. 2011).

Recently, Krall et al. (2013) reported on the association of 1-day average concentrations of species from the speciation network and mortality in 72 cities for the years 2000–2005. In this paper we address a similar question, but with the following differences. First, Krall et al. (2013) analyzed PM components without controlling for PM mass risks. As pointed out by Mostofsky et al. (2012), it is possible to find associations for components because they are highly correlated with mass, and not because they are themselves particularly toxic. Second, Krall et al. (2013) focused on single-day exposures. PM_2.5_ mortality studies have consistently reported that the associations are spread over > 1 day. Thus, when one uses separate time series for components that are measured only 1 day in 6 or 1 day in 3, this will bias downward estimates, possibly more for some components than others. In addition, the loss of two-thirds to five-sixths of the data substantially reduces power.

U.S. adults—particularly the elderly, who dominate mortality statistics—spend approximately 90% of their time indoors (U.S. EPA 1989). Although particles penetrate indoors, the infiltration rates vary with the extent to which windows and doors are open, which in turn can vary with local temperature and may therefore modify the association. Previous studies have reported such modification (Franklin et al. 2008; Stafoggia et al. 2008; Zanobetti et al. 2009). In this paper we address these issues and also examine more species, add an additional year of observation, and look at specific causes of death.

## Materials and Methods

*Study sites*. We included 75 U.S. cities in our study (see Supplemental Material, Table S1). Cities of interest were selected based on the availability of daily mortality, PM_2.5_ mass, and speciation data for at least 400 days between 2000 and 2006.

*Environmental data*. We conducted county-level analysis for most cities because the city lies within a single county, and used multiple counties for a city whose population extends beyond the boundary of one county (Zanobetti and Schwartz 2009). We obtained PM_2.5_ mass and species concentration data from the U.S. EPA Air Quality System Technology Transfer Network (http://www.epa.gov/ttn/airs/airsaqs/). PM_2.5_ mass samples were collected daily in most of the cities, whereas the speciation monitoring sites were operated on a 1-in-3 or 1-in-6 day schedule. Most of the cities had a single monitor. For cities with more than one sampling site, concentration data were averaged. Our analysis focused on organic carbon (OC), elemental carbon (EC), sodium (Na), aluminum (Al), silicon (Si), sulfur (S), potassium (K), calcium (Ca), vanadium (V), iron (Fe), nickel (Ni), copper (Cu), and zinc (Zn) because these species have been shown to be representative of several sources (e.g., motor vehicles, oil combustion, coal combustion, wood burning, sea salt, and road dust) and their concentration levels are mostly above the method detection limits (Hopke et al. 2006). Furthermore, they have been studied by previous epidemiologic and toxicological studies (Bell et al. 2009; Franklin et al. 2008; Ostro et al. 2006; Zanobetti et al. 2009; Zhou et al. 2011). Monthly average proportions between each component and PM_2.5_ mass were calculated for each city by averaging over the proportions of species to mass in each month, respectively.

Daily mean temperature in every city was obtained from the National Oceanic and Atmospheric Administration (http://www.noaa.gov/). We used 24-hr average temperature data from the closest weather station to the center of the city. Percent green space data were obtained from the National Land Cover Database, Multi-Resolution Land Characteristics Consortium (http://www.mrlc.gov/).

*Health data*. Daily mortality data were obtained from National Center for Health Statistics (http://www.cdc.gov/nchs/). We examined nonaccidental deaths due to all causes and specific diseases, which were derived from the *International Statistical Classification of Disease, 10th Revision* (World Health Organization 2007) codes as follows: all causes (ICD-10, A00–R99), cardiovascular diseases (ICD-10, I01–I59), respiratory diseases (ICD-10, J00–J99), myocardial infarction (ICD-10, I21–I22), and stroke (ICD-10, I60–I69).

We investigated several behavioral and other risk factors that have been reported to impact health (Baja et al. 2010; Dogra et al. 2007; Dwyer-Lindgren et al. 2013; Mora et al. 2007), including diabetes, being overweight or obese (i.e., body mass index ≥ 25), smoking, quitting smoking, alcohol consumption (having > two drinks per day), asthma, and leisure time physical activity, from the Behavioral Risk Factor Surveillance System (BRFSS) (Centers for Disease Control and Prevention 2006). We applied county-level weighting methodology to obtain county-level percentages of these variables in 2006. For counties that were not available, we used data from the closest metropolitan or micropolitan statistical area (MMSA) and applied MMSA-level weighting methodology.

*Statistical methods*. We applied a two-stage analysis in our study. In the first stage, we used a city-specific season-stratified time-series analysis using Poisson regression in a generalized additive model (GAM) to estimate the association between daily mortality and the mean of PM_2.5_ mass on the day of death and the day before death in each city and each season (defined as spring, March–May; summer, June–August; fall, September–November; winter, December–February). We controlled for time trend with a natural cubic regression spline with 1.5 degrees of freedom (df) per season per year, for day of the week with indicator variables, and for daily temperature on the same day (lag 0) and on the previous day (lag 1) with a natural cubic spline with 3 df for each. For every species, we calculated the monthly average species-to-PM_2.5_ proportions for each month as a solution to the missing speciation data problem due to the 1-in-6 or 1-in-3 day sampling frequency. We then added, one at the time, the interaction terms between PM_2.5_ and the monthly average species-to-PM_2.5_ proportions of each individual species (Valdes et al. 2012). The model is as below:

Log*E*(*Y_t_*) = Intercept + *ns*(time, *df*) + *ns*(temperature*_t_, df*) + *ns*(temperature*_t–_*_1_, *df*) + day of the week + α*Z_t–_*_1_*_,t_* + β*p^i^* + γ*Z_t–_*_1_*_,t_p^i^*, [1]

where, *E*(*Y_t_*) is the expected death count at day *t*, *ns* is the natural cubic splines, *Z_t–_*_1_*_,t_* indicates 2-day averaged concentration of PM_2.5_ at day *t–*1 and *t*, and *p^i^* is the mean monthly proportion of species *i* to mass.

By using an interaction with the monthly mean ratio, we avoided losing most of the daily observations, because we were able to use more than 1 day’s exposure, and control for PM mass. Although the use of the monthly ratio introduces some error in that variable, much of the variation in species mass is across cities, and between months within cities. For example, OC, sulfate, and nitrate are products of photochemical reactions whose rates are temperature dependent, and this varies substantially across the United States and differently by month in different locations (Baker and Scheff 2007; de Gouw et al. 2005). If a species ratio is not significant in this analysis, that does not mean that the species has no effect; it means its effect is not different from the average PM effect. A species with low or no toxicity would be expected to have a significant negative interaction term.

In the second stage of the analysis, we conducted a multivariate random effects meta-analysis and combined the 300 (i.e., 75 cities × 4 seasons) city–season specific effect estimates to obtain an overall association between PM_2.5_ mass and its interaction with each species with mortality across all 75 cities:

*Y^i^* = *XB^i^*, [2]

where, *Y^i^* is a (300 × 2) matrix, whose first column contains 300 city–season specific coefficients for PM_2.5_ and the second column contains 300 city–season specific coefficients for interaction with species *i*; *X* is a (300 × 4) matrix for intercept, linear, quadratic, and cubic temperature; and *B^i^* indicates a (4 × 2) matrix of meta-regression coefficient for PM_2.5_ and for interaction with species *i*.

It has been shown that high ventilation is seen at mild temperatures whereas low ventilation is seen at high and low temperatures (Koutrakis et al. 2005). Assuming that PM effect would not drop consistently as temperature increases, we added a cubic term in the model to allow for a plateau. We also examined whether the BRFSS factors modified PM_2.5_ effects. The model is:

β̂*_is_ =* β_0_ *+* β_1_*t_is_ +* β_2_*t_is_*^2^ *+* β_3_*t_is_*^3^ *+* β_4_*BRFSS_i_*, [3]

where β̂*_is_* is the estimated PM_2.5_ coefficient for city *i* in season *s*, *t_is_* is the centered temperature (i.e., temperature – mean temperature) for city *i* in season *s*, and *BRFSS_i_* is the BRFSS variable in city *i*. To estimate the effect of individual species, we performed the same meta-regression, but with the coefficient of the interaction term for species as the outcome being modeled. Here again we adjusted for city–season mean temperature as a surrogate for air exchange. We also investigated spatial variations between cities by focusing on a single outcome and exposure season to evaluate the effects in each city by mean exposure in that season for each city.

The effect estimates for PM_2.5_ were expressed as the percent change in mortality associated with a 10-μg/m^3^ increase in the 2-day averaged concentration of PM_2.5_ mass, for comparability with most previous studies. We expressed the effect of species on mortality as the estimated percent increase in mortality at the 10th and 90th percentile of distribution of species-to-PM_2.5_ proportion for each species, holding the PM_2.5_ increase constant at 10 μg/m^3^.

Data management was performed with SAS version 9.1 (SAS Institute Inc., Cary, NC), and regression analysis with R version 3.0.0 (http://www.r-project.org/).

## Results

In this study, we examined 4,473,519 all-cause deaths, of which 1,429,968 were cardiovascular disease (CVD), 308,235 mycardial infarction (MI), 255,430 stroke, and 436,800 respiratory deaths.

Table 1 summarizes daily mortality, PM_2.5_, and temperature in all cities. On average, there were 28 nonaccidental deaths per day. Daily death count by season was higher in the winter (*n* = 31) and spring (*n* = 28). Among the causes of interest, CVD killed the most people on average (9/day), followed by respiratory diseases (3/day). The overall mean concentration of PM_2.5_ was 13.3 μg/m^3^. PM_2.5_ mean concentration was highest in the summer (15.0 μg/m^3^) and lowest in the spring (11.6 μg/m^3^). Some of the species exhibited strong seasonal variability. For example, sulfur varied from 798 ng/m^3^ in the winter to 1,669 ng/m^3^ in the summer, with larger variations in some cities.

**Table 1:** Summary (mean ± SD) of daily mortality counts, PM_2.5_, and temperature across all 75 cities in 2000–2006.

Variable	Overall	Spring	Summer	Fall	Winter
Mortality (*n*)
All causes	28.0 ± 33.9	28.2 ± 33.9	26.0 ± 31.4	27.1 ± 32.5	30.8 ± 37.2
CVD	9.0 ± 12.6	9.1 ± 12.6	8.2 ± 11.5	8.5 ± 11.8	10.0 ± 14.1
MI	1.9 ± 2.9	1.9 ± 2.9	1.8 ± 2.6	1.8 ± 2.7	2.2 ± 3.3
Stroke	1.6 ± 2.2	1.6 ± 2.2	1.5 ± 2.0	1.6 ± 2.1	1.8 ± 2.4
Respiratory diseases	2.7 ± 3.6	2.9 ± 3.7	2.3 ± 3.1	2.4 ± 3.2	3.3 ± 4.4
Temperature (°C)	14.1 ± 10.0	13.4 ± 7.5	24.0 ± 4.1	15.1 ± 7.3	3.6 ± 8.0
PM_2.5_ (μg/m^3^)	13.3 ± 8.3	11.6 ± 6.5	15.0 ± 8.8	12.8 ± 8.4	13.9 ± 9.0
PM_2.5_ species^*a*^ (ng/m^3^)
OC	4,367 ± 2,752	3,688 ± 1,806	4,590 ± 2,371	4,491 ± 2,716	4,688 ± 3,724
EC	724 ± 590	602 ± 438	628 ± 459	830 ± 647	842 ± 733
Na	80 ± 141	93 ± 165	89 ± 152	66 ± 117	71 ± 122
Al	31 ± 78	31 ± 55	51 ± 128	23 ± 45	15 ± 34
Si	117 ± 177	123 ± 134	171 ± 273	98 ± 125	69 ± 85
S	1,174 ± 1,019	1,066 ± 731	1,669 ± 1,385	1,107 ± 960	798 ± 512
K	79 ± 197	63 ± 49	103 ± 360	69 ± 62	79 ± 103
Ca	65 ± 77	65 ± 68	74 ± 77	68 ± 88	53 ± 72
V	2.5 ± 4.0	2.2 ± 3.4	2.7 ± 4.2	2.7 ± 4.4	2.5 ± 3.8
Fe	102 ± 124	93 ± 127	111 ± 111	108 ± 136	93 ± 121
Ni	2.5 ± 11.6	2.3 ± 6.0	2.2 ± 6.5	2.2 ± 5.7	3.2 ± 21.4
Cu	5.1 ± 8.9	4.2 ± 7.3	5.7 ± 11.5	5.0 ± 7.3	5.4 ± 8.6
Zn	18 ± 57	16 ± 39	16 ± 57	19 ± 53	22 ± 76
^***a***^Method detection limits for species are available online (https://aqs.epa.gov/aqsweb/codes/data/Parameters-SPECIATION.csv).					

The distributions of monthly average proportions of PM_2.5_ species are shown in Table 2. OC had the largest mean proportion (37.9%), followed by sulfur (8.78%) and EC (6.31%). The mean proportions for all the metals were < 1% of mass concentration.

**Table 2:** Distributions of monthly species-to-PM_2.5_ proportions (%) across all 75 cities.

Species	Mean ± SD	Percentile				
		10th	25th	50th	75th	90th
OC	37.90 ± 16.90	24.60	29.10	35.50	44.10	53.60
EC	6.31 ± 3.45	2.86	3.96	5.51	7.49	10.10
Na	0.82 ± 1.31	0.07	0.20	0.45	0.96	1.90
Al	0.28 ± 0.44	0.03	0.07	0.15	0.31	0.64
Si	1.07 ± 1.22	0.30	0.45	0.70	1.20	2.12
S	8.78 ± 3.80	4.54	6.83	8.96	11.10	12.70
K	0.64 ± 0.52	0.31	0.40	0.53	0.72	0.98
Ca	0.62 ± 0.67	0.17	0.26	0.44	0.73	1.24
V	0.02 ± 0.03	0.00	0.01	0.01	0.03	0.05
Fe	0.89 ± 0.72	0.33	0.47	0.70	1.07	1.57
Ni	0.02 ± 0.06	0.00	0.00	0.01	0.02	0.04
Cu	0.04 ± 0.05	0.01	0.02	0.03	0.05	0.08
Zn	0.15 ± 0.18	0.04	0.06	0.10	0.15	0.24

Table 3 presents the estimated percent increase mortality for a 10-μg/m^3^ increase in 2-day averaged PM_2.5_ across the 75 cities. We found statistically significant associations between PM_2.5_ and mortality. A 1.18% (95% CI: 0.93, 1.44%) increase in all-cause mortality was associated with a 10-μg/m^3^ increase in the 2-day averaged concentration of PM_2.5_. The greatest effect estimate effect was observed for stroke mortality (1.76%; 95% CI: 1.01, 2.52%), followed by respiratory deaths (1.71%; 95% CI: 1.06, 2.35%). We observed seasonal variations in PM_2.5_ effects (see Supplemental Material, Figure S1). For a 10-μg/m^3^ increase in 2-day averaged PM_2.5_, the percent increases in all mortality categories were greatest in the spring.

**Table 3:** Estimated percent difference in mortality (95% CI) in association with a 10-μg/m^3^ increase in PM_2.5_ at lag 0–1 by cause of death and season.

Mortality	Overall	Spring	Summer	Fall	Winter
All causes	1.18 (0.93, 1.44)	2.85 (2.23, 3.47)	0.85 (0.42, 1.28)	1.17 (0.72, 1.63)	0.46 (0.07, 0.85)
CVD	1.03 (0.65, 1.41)	2.47 (1.52, 3.43)	1.03 (0.38, 1.67)	0.87 (0.33, 1.42)	0.39 (–0.36, 1.14)
MI	1.22 (0.62, 1.82)	2.08 (0.72, 3.46)	1.23 (–0.19, 2.66)	0.81 (–0.32, 1.95)	0.41 (–1.12, 1.96)
Stroke	1.76 (1.01, 2.52)	3.31 (0.49, 6.22)	1.16 (–0.42, 2.76)	1.31 (0.05, 2.58)	1.59 (0.16, 3.03)
Respiratory diseases	1.71 (1.06, 2.35)	4.03 (2.85, 5.21)	1.09 (–0.58, 2.78)	0.58 (–0.39, 1.57)	0.86 (–0.11, 1.84)

**Figure 1:**
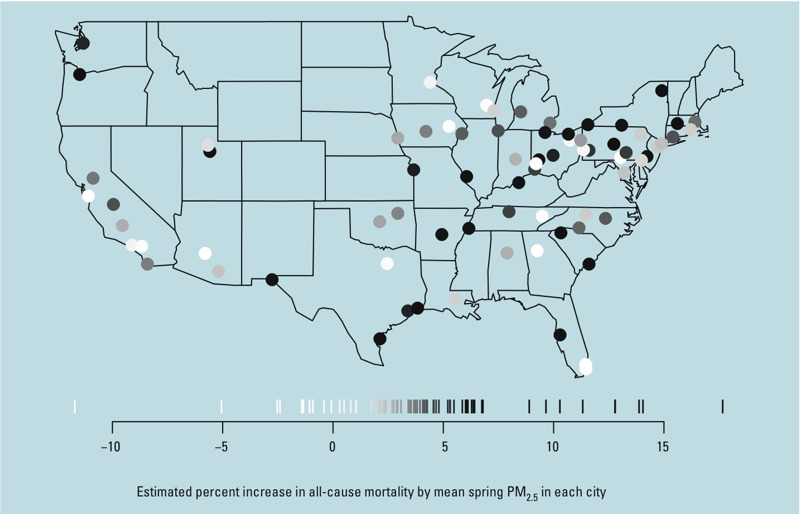
Spatial variations in estimated PM_2.5_ effects between cities.

Figure 1 shows the effect estimates of PM_2.5_ on all-cause mortality in each city by mean spring PM_2.5_ in each city. We observed differential effects across cities.

**Figure 2:**
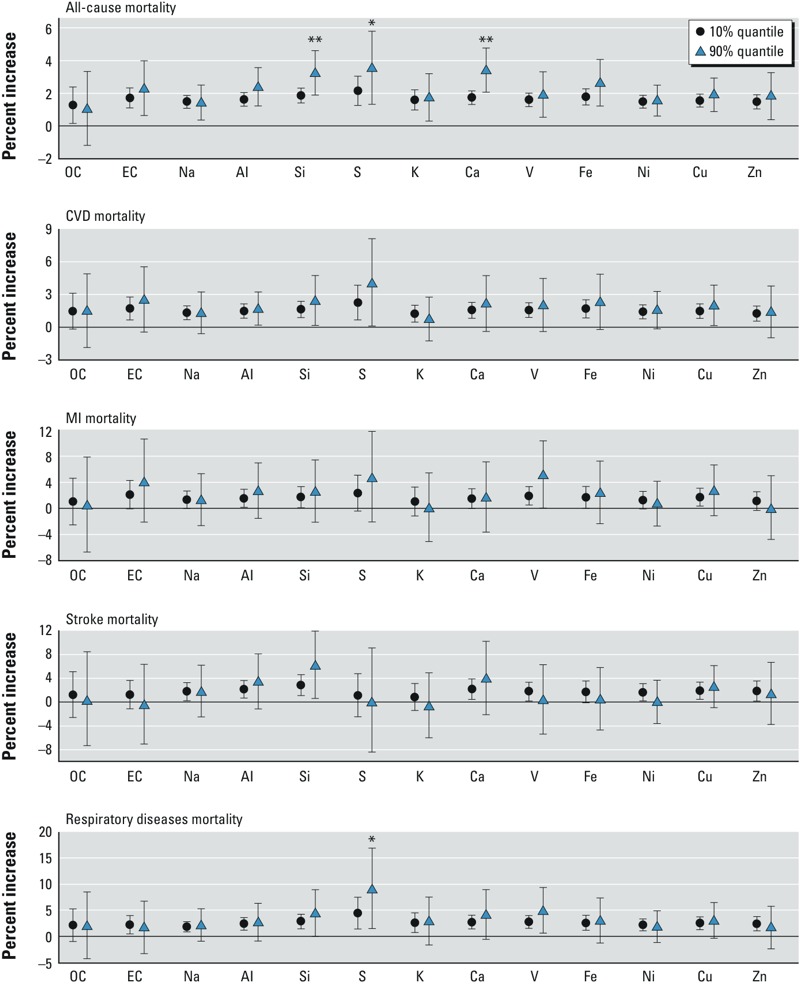
Estimated percent difference in mortality for a 10-μg/m^3^ increase in PM_2.5_ at lag 0–1 and an increase of 10th or 90th percentile of distribution of monthly species-to-PM_2.5_ proportions, controlled for city–season specific temperature. *p ≤ 0.1 for the interaction term. **p ≤ 0.05 for the interaction term.

Figure 2 shows the adjusted estimated percent increases in mortality for a 10-μg/m^3^ increase in 2-day averaged PM_2.5_ at the 10th or 90th percentile of distribution of the proportions of species. For all-cause mortality, interaction terms between PM_2.5_ and species silicon, calcium, and sulfur had a *p*-value ≤ 0.1. We found that a 10-μg/m^3^ increase in 2-day averaged PM_2.5_ was associated with an increase in all-cause mortality of 3.55% (95% CI: 1.35, 5.81%) at the 90th percentile of distribution of the sulfur-to-PM_2.5_ proportion versus 2.16% (95% CI: 1.27, 3.06%) at the 10th percentile of the sulfur-to-PM_2.5_ ratio (see Supplemental Material, Table S2). We also found that silicon (3.25%; 95% CI: 1.91, 4.62% vs. 1.87%; 95% CI: 1.42, 2.32%) and calcium (3.42%; 95% CI: 2.08, 4.77% vs. 1.75%; 95% CI: 1.34, 2.16%) were associated with higher estimated effects of PM_2.5_ on all-cause mortality. In addition, sulfur was associated with higher estimated PM_2.5_ effect on respiratory deaths. The percent increase in respiratory mortality at the 90th percentile of the sulfur-to-PM_2.5_ proportion was 8.96% (95% CI: 1.55, 16.90%), vs. 4.44% (95% CI: 1.46, 7.51%) at the 10th percentile.

**Figure 3:**
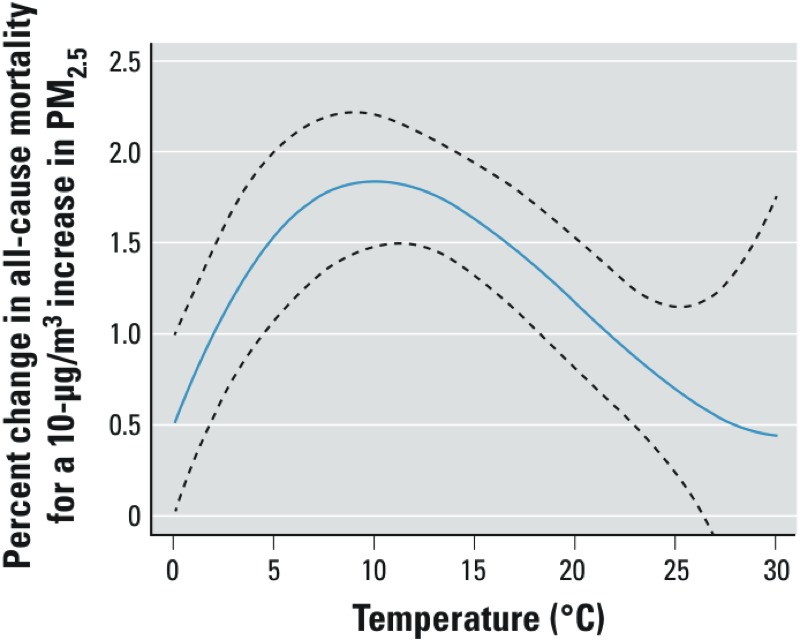
Relationship between estimated effects of PM_2.5_ on all-cause mortality and temperature (controlled for smoking and alcohol consumption).

Figure 3 indicates the relationship between effect estimates and city–season temperature, which serves as a surrogate for ventilation and thus particle penetration indoors. We observed an inverted U-shape relationship with a plateau at high temperatures. The *p*-value for cubic term is 0.06 in meta-regression without BRFSS factors and is 0.07 controlled for smoking and alcohol consumption. The all-cause mortality effect estimates first increase as temperature increases and peak around a seasonal average of 10°C. After that, they decrease until they reach a plateau at around 28°C.

County-level percent of green space, diabetes, obesity, asthma, or physical activity did not modify the effect of PM_2.5_ on mortality. However, among the behavioral factors, we found the effects of PM_2.5_ were higher in areas where people smoked more or had two drinks or more per day. Specifically, an interquartile range (IQR) increase in the prevalence of smokers (8.8%) was associated with a 34% increase in estimated PM_2.5_ effects, whereas an IQR increase in the prevalence of heavy drinkers (7.0%) was associated with an increase of 40% in the estimated effects of fine particles.

## Discussion

In this nationwide time-series study, we estimated the effects of PM_2.5_ mass and species on daily mortality across 75 U.S. cities, covering > 4 million deaths. We found that an increase in PM_2.5_ concentration at lag day 0–1 was statistically significantly associated with increased risk of all-cause mortality, CVD, MI, stroke, and respiratory mortality. We also found that PM_2.5_-related effects were modified by certain species. Furthermore, analysis by season indicated that effect estimates were highest in the spring. To investigate this seasonal pattern we included city–season specific temperature in the meta-regression analysis. These seasonal variations may affect the characteristics of PM_2.5_ mixture and mediate its effects on health outcomes (Bell et al. 2007).

Controlling for this potential confounder and for PM_2.5_ mass, we found that a species related to coal combustion (i.e., sulfur) was associated with higher risks for all-cause but particularly respiratory mortality. Sulfur is also a marker of regional pollution; thus, it may not reflect only exposures to power plant emissions. Changes in the proportion of OC mass in PM_2.5_ did not modify its effect on mortality for any cause, suggesting this species has average toxicity. We found that higher silicon or calcium proportions were associated with increased estimated PM_2.5_ mortality risks. These crustal elements are often elevated near roads and can be a surrogate for increased road dust, which in addition to those elements contains various organic compounds, compounds from tire and brake wear, and the like (Rogge et al. 1993). Thus they may be a marker for pollution from traffic other than EC.

The BRFSS factors we examined were on the county level. The distributions of prevalence of smoking and heavy drinkers (i.e., > two drinks/day) in different cities were approximately normal distributed with a mean around 30% and 60%, respectively (see Supplemental Material, Figure S2). We found that cities with more smokers or heavy drinkers had larger estimated effects of PM_2.5_. These have not previously been identified as susceptibility factors for the effects of particles on health, and this requires greater attention.

The magnitudes of effects in our study are comparable with those reported by other studies. For example, a study that included 112 U.S. cities reported a 0.98% (95% CI: 0.75, 1.22%) increase, a 0.85% (95% CI: 0.46, 1.24%) increase, a 1.18% (95% CI: 0.48, 1.89%) increase, a 1.78% (95% CI: 0.96. 2.62%) increase, and a 1.68% (95% CI: 1.04, 2.33%) increase in all-cause, CVD, MI, stroke, and respiratory mortality, respectively, for a 10-μg/m^3^ increase in 2-day averaged PM_2.5_ (Zanobetti and Schwartz 2009). Our estimates are slightly higher than the ones above and are closer to those by a 27-city study, which found a 1.21% (95% CI: 0.29, 2.14%) increase in all-cause mortality, a 1.78% (95% CI: 0.20, 3.36%) increase in respiratory mortality, and a 1.03% (95% CI: 0.02, 2.04%) increase in stroke mortality for a 10-μg/m^3^ increase in previous day’s PM_2.5_ (Franklin et al. 2007). Our study and these two studies used city–season specific models to allow for seasonal differences in the effects of temperature and day of the week.

The finding that effects were highest in the spring is consistent with previous studies (Zanobetti and Schwartz 2009; Zeka et al. 2006). Franklin et al. (2008) found similar pattern using linear and quadratic temperature in the meta-regression. Additionally, we included the cubic term, which was marginally significant and led to the small plateau at high temperatures. These results indicated greater effects for moderate temperatures when windows are more likely to be open and particle penetration rates are higher.

EC is considered a marker of traffic emissions (Viana et al. 2006). Previous research has reported that EC was significantly associated with increased risk of mortality due to all causes or CVD (Bell et al. 2009; Metzger et al. 2004; Peng et al. 2009). In this study, we did observe that increase in the EC-to-PM_2.5_ proportion increased the association between PM_2.5_ and all-cause mortality and CVD mortality in crude meta-regression, but it was no longer significant when we controlled for city–season temperature. Similarly, two studies also controlled for temperature in the meta-regression and did not find any effect modification by EC in the association between PM_2.5_ and nonaccidental mortality or hospital admissions for cardiovascular diseases (Franklin et al. 2008; Zanobetti et al. 2009).

Silicon and calcium, which may be associated with soil or road dust, modified the effects of PM_2.5_ on all-cause mortality in our study. Crustal elements have been reported to have adverse effects on health. For example, Ostro et al. (2010) found strong association between silicon and mortality; Franklin et al. (2008) observed that silicon and aluminum were modifiers of the PM_2.5_–mortality effects. Other studies have shown plausible biological mechanisms of inflammatory effects of road dust containing aluminum and/or silicon (Becker et al. 2005; Clarke et al. 2000). Additionally, road dust is often coated with organic compounds and metals from car exhaust, tire wear, and the like (Rogge et al. 1993), which may contribute to its toxicity.

Nickel, as a marker of oil combustion, was reported to have effect modification in the relationship between PM_2.5_ and mortality or hospital admissions in previous studies (Franklin et al. 2008; Zanobetti et al. 2009), but we did not observe any. On average, nickel accounted for only 0.02% of the PM_2.5_ concentration in our study. The concentrations of nickel are frequently lower than the method detection limit (Burnett et al. 2000), which may make us fail to detect its effects. Nevertheless, toxicological research has found evidence on its adverse effects (Gao et al. 2004; Lippmann et al. 2006). For example, Lippmann et al. (2006) found that atherosclerotic-prone mice exposed to concentrated air particles had a pronounced acute change in heart rate and heart rate variability when nickel was especially high. Lippmann et al. (2006) found that nickel effects had high exposures due to the residual fuel burn in New York City for heating. The levels for the entire country are lower.

We observed that the effect of PM_2.5_ mass on all-cause and respiratory mortality was modified by sulfur. This component is a marker of coal combustion emissions, which suggests that species derived from coal combustion might have greater toxicity effects on mortality, particularly due to respiratory diseases. Sulfate is the primary form of sulfur in particles. Sulfate has been implicated as a major toxic species in PM_2.5_ (Amdur 1996) and reported to be associated with increased risk of various mortality outcomes in earlier epidemiological studies (Fairley 1999; Hoek et al. 2000; Laden et al. 2000; Mar et al. 2000). The importance of sulfates in the air may be attributable to the ability of acid sulfates to solubilize transition metals, thus making them bioavailable (Ghio et al. 1999). Studies have found that sulfate was associated with endothelial dysfunction (O’Neill et al. 2005), increased oxidative stress, and coagulation (Chuang et al. 2007). These toxicology findings provide plausibility to sulfate health effects.

One disappointing aspect of this result is that despite the use of 75 cities and almost 4.5 million deaths, we were unable to distinguish much difference in toxicity for many of the species we examined. This may reflect only modest differences in toxicity, but may also reflect more fundamental difficulties in identifying differences between many correlated exposures with limited measurements over time. Evidence of the low power to detect differences can be seen in the difference between our results for all deaths and results for CVD deaths. The pattern of higher estimated effects when PM mass has a larger fraction of silicon, sulfur, and calcium is present for CVD deaths as well; but with one-third as many deaths, it does not reach significance. One option to improve study power might be specifically selecting locations with high proportions of the species of interest.

There are several limitations in this study. First, our ability to capture spatial variability is constrained by the location of U.S. EPA monitors. A previous study showed moderate to low monitor-to-monitor correlations between daily concentrations of several species (arsenic, EC, and nickel) in the New York City area, which suggest high spatial variability in some speciation concentrations (Ito et al. 2004). Differential measurement error between species that are better or worse represented by a single monitor may bias differential results. However, in a time-series study much of the geographic variability will result in Berkson error (Zeger et al. 2000), which will not produce bias. Meanwhile, failing to capture spatial variability might weaken study power and attenuate estimates. Second, we failed to capture day-to-day variation in the analysis. Although we used monthly average species-to-PM_2.5_ proportions to gain more power, we still lost variation across days.

Third, as mentioned above, there are data limitations, such as the one-in-six and one-in-three sampling frequency for the species. Whether one takes the approach of Krall et al. (2013) and analyzes only those days, or takes our approach and gains power by analyzing every day but with more error-prone monthly means of the species, there is a price paid for this lack of data. Together with moderate correlation among the species and with total particle mass, this makes the task difficult. We do not believe this is likely to produce false positives, and hence we believe our findings are well supported.

## Supplemental Material

(487 KB) PDF
