# Erratum: “Associations of Fine Particulate Matter Species with Mortality in the United States: A Multicity Time-Series Analysis”

**DOI:** 10.1289/EHP301

**Published:** 2016-06-01

**Authors:** Lingzhen Dai, Antonella Zanobetti, Petros Koutrakis, Joel D. Schwartz

Environ Health Perspect 122(8):837–842 (2014), http://dx.doi.org/10.1289/ehp.1307568

In Table S1, the authors incorrectly inverted the titles of the column headers for temperature and PM_2.5_. In the original supplemental materials, the last two column headings were titled Temperature (°C) and PM_2.5_ (µg/m^3^), but should have been PM_2.5_ (µg/m^3^) and Temperature (°C), respectively. The authors also noticed the following misspelling: Saint Diego, CA, should be spelled San Diego, CA. These corrections to Table S1 appear in this erratum.

These typographical errors do not affect the information presented in the article. The authors regret the errors.

**Table S1:** City specific summary (mean ± SD) of all-cause mortality, PM_2.5_, and temperature, 2000–2006.

#	City	All-cause mortality per 100,000 in population (No.)	PM_2.5_ (µg/m^3^)	Temperature (°C)
1	Akron, OH	2.6 ± 0.7	15.8 ± 8.4	10.0 ± 10.1
2	Atlanta, GA	1.6 ± 0.3	16.5 ± 7.4	17.0 ± 8.0
3	Bakersfield, CA	1.9 ± 0.6	16.6 ± 14.0	18.7 ± 7.7
4	Bath, NY	2.0 ± 1.1	9.4 ± 6.7	8.9 ± 10.2
5	Birmingham, AL	2.9 ± 0.7	15.8 ± 8.1	17.4 ± 8.4
6	Boston, MA	2.3 ± 0.4	11.9 ± 6.7	10.9 ± 9.5
7	Baton Rouge, LA	2.0 ± 0.7	13.2 ± 6.0	20.1 ± 7.4
8	Cedar Rapids, IA	3.0 ± 1.5	11.0 ± 7.2	9.4 ± 11.4
9	Charlotte, NC	1.7 ± 0.5	14.9 ± 6.8	15.7 ± 8.3
10	Charleston, SC	5.7 ± 2.2	12.0 ± 5.7	18.9 ± 7.6
11	Chicago, IL	2.5 ± 0.3	15.2 ± 8.2	10.3 ± 10.6
12	Cincinnati, OH	2.5 ± 0.6	16.8 ± 8.2	12.4 ± 9.7
13	Cleveland, OH	2.7 ± 0.4	15.2 ± 8.8	10.5 ± 10.0
14	Columbus, OH	2.0 ± 0.5	16.1 ± 8.3	11.8 ± 10.1
15	Corpus Christ, TX	2.0 ± 0.8	10.2 ± 4.1	23.4 ± 5.8
16	Dallas, TX	1.6 ± 0.3	12.5 ± 5.8	19.4 ± 8.8
17	Davenport, IA	4.3 ± 1.6	12.2 ± 7.1	11.1 ± 11.1
18	Dayton, OH	2.5 ± 0.7	16.2 ± 8.3	11.2 ± 10.3
19	Des Moines, IA	1.9 ± 0.7	10.3 ± 6.4	10.9 ± 11.4
20	Detroit, MI	2.2 ± 0.3	15.4 ± 9.1	10.4 ± 10.4
21	Dodge, WI	2.8 ± 1.6	10.9 ± 7.6	8.2 ± 11.6
22	Elizabeth, NJ	2.2 ± 0.7	14.4 ± 8.6	13.1 ± 9.7
23	El Paso, TX	1.5 ± 0.5	10.0 ± 5.1	18.5 ± 8.8
24	Erie, PA	2.5 ± 1.0	12.8 ± 8.1	10.1 ± 9.9
25	Essex, NY	0.3 ± 0.2	6.2 ± 5.5	6.0 ± 11.0
26	Fresno, CA	1.8 ± 0.5	19.0 ± 15.3	18.3 ± 7.7
27	Fort Lauderdale, FL	2.4 ± 0.4	8.4 ± 4.0	22.8 ± 4.9
28	Gettysburg, PA	2.5 ± 1.3	13.2 ± 8.3	11.5 ± 9.7
29	Grand Rapids, MI	1.1 ± 0.3	13.6 ± 8.7	9.1 ± 10.5
30	Greenville, SC	2.5 ± 1.3	15.0 ± 6.9	16.2 ± 8.1
31	Harrisburg, PA	2.5 ± 1.0	15.4 ± 9.3	12.1 ± 9.8
32	Houston, TX	1.5 ± 0.2	12.8 ± 5.5	21.1 ± 7.3
33	Indianapolis, IN	2.2 ± 0.5	16.2 ± 8.2	12.0 ± 10.3
34	Kansas City, KS	2.5 ± 0.5	11.9 ± 6.0	13.2 ± 10.7
35	Knoxville, TN	3.1 ± 0.9	15.3 ± 7.1	15.3 ± 8.7
36	Los Angeles, CA	1.6 ± 0.2	17.8 ± 10.3	17.2 ± 3.4
37	Louisville, KY	2.5 ± 0.7	15.6 ± 7.9	14.6 ± 9.7
38	Little Rock, AR	2.3 ± 0.8	13.9 ± 6.7	17.2 ± 9.1
39	Memphis, TN	2.1 ± 0.5	13.2 ± 6.6	17.5 ± 9.0
40	Miami, FL	2.1 ± 0.3	9.1 ± 4.3	25.0 ± 3.7
41	Middletown, OH	2.1 ± 0.8	16.0 ± 8.1	9.8 ± 10.4
42	Milwaukee, WI	3.1 ± 0.6	13.4 ± 8.1	9.2 ± 10.5
43	Minneapolis, MN	1.9 ± 0.4	11.6 ± 7.3	8.4 ± 12.2
44	Nashville, TN	2.2 ± 0.6	13.9 ± 6.7	15.7 ± 9.0
45	New Haven, CT	3.8 ± 0.9	13.4 ± 8.1	10.4 ± 10.1
46	New York City, NY	1.8 ± 0.2	14.5 ± 8.4	13.4 ± 9.6
47	Oklahoma City, OK	2.4 ± 0.7	9.9 ± 5.2	17.2 ± 9.4
48	Omaha, NE	2.0 ± 0.7	10.3 ± 6.0	11.2 ± 11.5
49	Port Arthur, TX	11.3 ± 4.5	11.1 ± 5.5	20.8 ± 7.1
50	Philadelphia, PA	7.4 ± 1.9	14.1 ± 8.2	13.5 ± 9.6
51	Phoenix, AZ	1.8 ± 0.3	11.2 ± 7.1	24.1 ± 8.9
52	Pittsburgh, PA	3.0 ± 0.6	15.6 ± 10.1	10.9 ± 9.8
53	Portland, OR	4.2 ± 0.9	8.8 ± 6.0	12.2 ± 6.2
54	Providence, RI	5.1 ± 1.1	10.9 ± 6.5	11.0 ± 9.5
55	Provo, UT	3.7 ± 1.8	9.5 ± 8.7	12.0 ± 10.5
56	Raleigh, NC	1.4 ± 0.5	14.1 ± 6.6	15.8 ± 8.7
57	Riverside, CA	4.1 ± 0.7	17.4 ± 11.4	19.1 ± 5.8
58	Sacramento, CA	2.0 ± 0.4	12.3 ± 10.3	16.3 ± 6.4
59	Salt Lake City, UT	1.4 ± 0.4	11.3 ± 10.9	11.9 ± 10.5
60	State College, PA	1.8 ± 1.0	12.9 ± 8.4	10.4 ± 9.8
61	Scranton, PA	8.7 ± 2.2	12.0 ± 7.9	10.0 ± 10.0
62	San Diego, CA	1.8 ± 0.3	12.6 ± 7.3	17.6 ± 3.3
63	Seattle, WA	1.7 ± 0.3	9.4 ± 5.8	11.4 ± 5.6
64	San Jose, CA	1.3 ± 0.3	13.2 ± 11.0	16.4 ± 4.7
65	Springfield, MA	2.7 ± 0.8	12.3 ± 7.6	10.2 ± 10.1
66	Saint Louis, MO	2.4 ± 0.5	14.0 ± 7.2	14.2 ± 10.4
67	Tampa, FL	2.2 ± 0.5	11.3 ± 5.1	23.0 ± 5.4
68	Toledo, OH	2.5 ± 0.8	14.9 ± 8.4	10.5 ± 10.4
69	Tucson, AZ	2.3 ± 0.6	6.1 ± 2.4	21.2 ± 7.8
70	Tulsa, OK	2.4 ± 0.7	11.3 ± 6.0	17.1 ± 9.7
71	Washington, PA	3.1 ± 1.3	14.6 ± 7.9	11.7 ± 9.7
72	Washington DC	1.9 ± 0.5	14.9 ± 8.2	14.5 ± 9.3
73	Wilmington, DE	2.0 ± 0.7	14.9 ± 8.3	12.6 ± 9.5
74	Winston, NC	2.3 ± 0.9	14.5 ± 7.4	15.2 ± 8.6
75	Youngstown, OH	2.9 ± 0.9	15.1 ± 8.0	9.6 ± 9.9

