# Supplementary material for: Associations of Fine Particulate Matter Species with Mortality in the United States: A Multicity Time-Series Analysis
Source: Environ Health Perspect. 2014 May 6;122(8):837–42. doi: 10.1289/ehp.1307568 (PMC4123030; doi:10.1289/ehp.1307568)

Supplemental Material

Associations of Fine Particulate Matter Species with Mortality in the United States: A Multicity Time-Series Analysis

Lingzhen Dai, Antonella Zanobetti, Petros Koutrakis, and Joel D. Schwartz

Table of Contents **Page**

Table S1. City specific summary (mean ± SD) of all-cause mortality, $PM_{2.5}$, and temperature, 2000-2006 **2**

Table S2. Estimated percent difference (95% CI) in mortality for a 10-μg/m^3 increase in $PM_{2.5}$ at lag 0-1 at the 10th or 90th percentile of the distribution of monthly species-to-$PM_{2.5}$ proportions, adjusted for city-season specific temperature **4**

Figure S1. Estimated percent difference in mortality in association with for a 10-μg/m^3 increase in $PM_{2.5}$ at lag 0-1 by cause of death and season **6**

Figure S2. Distribution of county-level percentage of smoking and alcohol consumption **7**

Table S1. City specific summary (mean ± SD) of all-cause mortality, $PM_{2.5}$, and temperature, 2000-2006.

#	City	All-cause mortality per 100,000 in population (no.)	Temperature (˚C)	$PM_{2.5}$ ($\mu g/m^3$)
1	Akron, OH	2.6 ± 0.7	15.8 ± 8.4	10.0 ± 10.1
2	Atlanta, GA	1.6 ± 0.3	16.5 ± 7.4	17.0 ± 8.0
3	Bakersfield, CA	1.9 ± 0.6	16.6 ± 14.0	18.7 ± 7.7
4	Bath, NY	2.0 ± 1.1	9.4 ± 6.7	8.9 ± 10.2
5	Birmingham, AL	2.9 ± 0.7	15.8 ± 8.1	17.4 ± 8.4
6	Boston, MA	2.3 ± 0.4	11.9 ± 6.7	10.9 ± 9.5
7	Baton Rouge, LA	2.0 ± 0.7	13.2 ± 6.0	20.1 ± 7.4
8	Cedar Rapids, IA	3.0 ± 1.5	11.0 ± 7.2	9.4 ± 11.4
9	Charlotte, NC	1.7 ± 0.5	14.9 ± 6.8	15.7 ± 8.3
10	Charleston, SC	5.7 ± 2.2	12.0 ± 5.7	18.9 ± 7.6
11	Chicago, IL	2.5 ± 0.3	15.2 ± 8.2	10.3 ± 10.6
12	Cincinnati, OH	2.5 ± 0.6	16.8 ± 8.2	12.4 ± 9.7
13	Cleveland, OH	2.7 ± 0.4	15.2 ± 8.8	10.5 ± 10.0
14	Columbus, OH	2.0 ± 0.5	16.1 ± 8.3	11.8 ± 10.1
15	Corpus Christ, TX	2.0 ± 0.8	10.2 ± 4.1	23.4 ± 5.8
16	Dallas, TX	1.6 ± 0.3	12.5 ± 5.8	19.4 ± 8.8
17	Davenport, IA	4.3 ± 1.6	12.2 ± 7.1	11.1 ± 11.1
18	Dayton, OH	2.5 ± 0.7	16.2 ± 8.3	11.2 ± 10.3
19	Des Moines, IA	1.9 ± 0.7	10.3 ± 6.4	10.9 ± 11.4
20	Detroit, MI	2.2 ± 0.3	15.4 ± 9.1	10.4 ± 10.4
21	Dodge, WI	2.8 ± 1.6	10.9 ± 7.6	8.2 ± 11.6
22	Elizabeth, NJ	2.2 ± 0.7	14.4 ± 8.6	13.1 ± 9.7
23	El Paso, TX	1.5 ± 0.5	10.0 ± 5.1	18.5 ± 8.8
24	Erie, PA	2.5 ± 1.0	12.8 ± 8.1	10.1 ± 9.9
25	Essex, NY	0.3 ± 0.2	6.2 ± 5.5	6.0 ± 11.0
26	Fresno, CA	1.8 ± 0.5	19.0 ± 15.3	18.3 ± 7.7
27	Fort Lauderdale, FL	2.4 ± 0.4	8.4 ± 4.0	22.8 ± 4.9
28	Gettysburg, PA	2.5 ± 1.3	13.2 ± 8.3	11.5 ± 9.7
29	Grand Rapids, MI	1.1 ± 0.3	13.6 ± 8.7	9.1 ± 10.5
30	Greenville, SC	2.5 ± 1.3	15.0 ± 6.9	16.2 ± 8.1
31	Harrisburg, PA	2.5 ± 1.0	15.4 ± 9.3	12.1 ± 9.8
32	Houston, TX	1.5 ± 0.2	12.8 ± 5.5	21.1 ± 7.3
33	Indianapolis, IN	2.2 ± 0.5	16.2 ± 8.2	12.0 ± 10.3
34	Kansas City, KS	2.5 ± 0.5	11.9 ± 6.0	13.2 ± 10.7
35	Knoxville, TN	3.1 ± 0.9	15.3 ± 7.1	15.3 ± 8.7
36	Los Angeles, CA	1.6 ± 0.2	17.8 ± 10.3	17.2 ± 3.4
37	Louisville, KY	2.5 ± 0.7	15.6 ± 7.9	14.6 ± 9.7

#	City	All-cause mortality per 100,000 in population (no.)	Temperature (˚C)	$PM_{2.5}$ (μg/m^3)
38	Little Rock, AR	2.3 ± 0.8	13.9 ± 6.7	17.2 ± 9.1
39	Memphis, TN	2.1 ± 0.5	13.2 ± 6.6	17.5 ± 9.0
40	Miami, FL	2.1 ± 0.3	9.1 ± 4.3	25.0 ± 3.7
41	Middletown, OH	2.1 ± 0.8	16.0 ± 8.1	9.8 ± 10.4
42	Milwaukee, WI	3.1 ± 0.6	13.4 ± 8.1	9.2 ± 10.5
43	Minneapolis, MN	1.9 ± 0.4	11.6 ± 7.3	8.4 ± 12.2
44	Nashville, TN	2.2 ± 0.6	13.9 ± 6.7	15.7 ± 9.0
45	New Haven, CT	3.8 ± 0.9	13.4 ± 8.1	10.4 ± 10.1
46	New York City, NY	1.8 ± 0.2	14.5 ± 8.4	13.4 ± 9.6
47	Oklahoma City, OK	2.4 ± 0.7	9.9 ± 5.2	17.2 ± 9.4
48	Omaha, NE	2.0 ± 0.7	10.3 ± 6.0	11.2 ± 11.5
49	Port Arthur, TX	11.3 ± 4.5	11.1 ± 5.5	20.8 ± 7.1
50	Philadelphia, PA	7.4 ± 1.9	14.1 ± 8.2	13.5 ± 9.6
51	Phoenix, AZ	1.8 ± 0.3	11.2 ± 7.1	24.1 ± 8.9
52	Pittsburgh, PA	3.0 ± 0.6	15.6 ± 10.1	10.9 ± 9.8
53	Portland, OR	4.2 ± 0.9	8.8 ± 6.0	12.2 ± 6.2
54	Providence, RI	5.1 ± 1.1	10.9 ± 6.5	11.0 ± 9.5
55	Provo, UT	3.7 ± 1.8	9.5 ± 8.7	12.0 ± 10.5
56	Raleigh, NC	1.4 ± 0.5	14.1 ± 6.6	15.8 ± 8.7
57	Riverside, CA	4.1 ± 0.7	17.4 ± 11.4	19.1 ± 5.8
58	Sacramento, CA	2.0 ± 0.4	12.3 ± 10.3	16.3 ± 6.4
59	Salt Lake City, UT	1.4 ± 0.4	11.3 ± 10.9	11.9 ± 10.5
60	State College, PA	1.8 ± 1.0	12.9 ± 8.4	10.4 ± 9.8
61	Scranton, PA	8.7 ± 2.2	12.0 ± 7.9	10.0 ± 10.0
62	Saint Diego, CA	1.8 ± 0.3	12.6 ± 7.3	17.6 ± 3.3
63	Seattle, WA	1.7 ± 0.3	9.4 ± 5.8	11.4 ± 5.6
64	San Jose, CA	1.3 ± 0.3	13.2 ± 11.0	16.4 ± 4.7
65	Springfield, MA	2.7 ± 0.8	12.3 ± 7.6	10.2 ± 10.1
66	Saint Louis, MO	2.4 ± 0.5	14.0 ± 7.2	14.2 ± 10.4
67	Tampa, FL	2.2 ± 0.5	11.3 ± 5.1	23.0 ± 5.4
68	Toledo, OH	2.5 ± 0.8	14.9 ± 8.4	10.5 ± 10.4
69	Tucson, AZ	2.3 ± 0.6	6.1 ± 2.4	21.2 ± 7.8
70	Tulsa, OK	2.4 ± 0.7	11.3 ± 6.0	17.1 ± 9.7
71	Washington, PA	3.1 ± 1.3	14.6 ± 7.9	11.7 ± 9.7
72	Washington DC	1.9 ± 0.5	14.9 ± 8.2	14.5 ± 9.3
73	Wilmington, DE	2.0 ± 0.7	14.9 ± 8.3	12.6 ± 9.5
74	Winston, NC	2.3 ± 0.9	14.5 ± 7.4	15.2 ± 8.6
75	Youngstown, OH	2.9 ± 0.9	15.1 ± 8.0	9.6 ± 9.9

Table S2. Estimated percent difference (95% CI) in mortality for a 10-μg/m^3 increase in $PM_{2.5}$ at lag 0-1 at the 10th or 90th percentile of the distribution of monthly species-to-$PM_{2.5}$ proportions, adjusted for city-season specific temperature.

Species	All causes	CVD	MI	Stroke	Respiratory diseases
**OC**					
10th percentile	1.28 (0.18, 2.39)	1.46 (-0.17, 3.12)	1.00 (-2.48, 4.61)	1.24 (-2.55, 5.17)	2.15 (-0.90, 5.30)
90th percentile	1.05 (-1.18, 3.34)	1.47 (-1.85, 4.89)	0.32 (-6.69, 7.86)	0.32 (-7.25, 8.52)	1.96 (-4.20, 8.52)
**EC**					
10th percentile	1.72 (1.12, 2.33)	1.71 (0.67, 2.77)	2.10 (-0.03, 4.28)	1.29 (-1.06, 3.69)	2.24 (0.49, 4.01)
90th percentile	2.31 (0.65, 4.00)	2.51 (-0.43, 5.54)	4.07 (-2.06, 10.58)	-0.53 (-7.00, 6.40)	1.63 (-3.24, 6.74)
**Na**					
10th percentile	1.49 (1.11, 1.87)	1.31 (0.69, 1.94)	1.33 (0.02, 2.65)	1.80 (0.30, 3.31)	1.85 (0.88, 2.82)
90th percentile	1.44 (0.37, 2.52)	1.30 (-0.60, 3.23)	1.28 (-2.62, 5.32)	1.80 (-2.45, 6.24)	2.16 (-0.87, 5.29)
**Al**					
10th percentile	1.63 (1.23, 2.04)	1.48 (0.84, 2.12)	1.54 (0.17, 2.93)	2.18 (0.68, 3.69)	2.45 (1.25, 3.65)
90th percentile	2.41 (1.25, 3.59)	1.70 (0.21, 3.23)	2.64 (-1.51, 6.97)	3.41 (-1.11, 8.13)	2.69 (-0.85, 6.35)
**Si**					
10th percentile	1.87 (1.42, 2.32)	1.63 (0.89, 2.38)	1.72 (0.14, 3.33)	2.87 (1.11, 4.66)	2.86 (1.50, 4.24)
90th percentile	3.25 (1.91, 4.62)	2.43 (0.16, 4.74)	2.57 (-2.06, 7.41)	6.17 (0.66, 11.97)	4.39 (0.03, 8.93)
**S**					
10th percentile	2.16 (1.27, 3.06)	2.25 (0.67, 3.84)	2.31 (-0.38, 5.09)	1.14 (-2.42, 4.82)	4.44 (1.46, 7.51)
90th percentile	3.55 (1.35, 5.81)	4.05 (0.13, 8.12)	4.63 (-2.07, 11.80)	0.03 (-8.34, 9.17)	8.96 (1.55, 16.90)
**K**					
10th percentile	1.60 (0.99, 2.22)	1.22 (0.45, 2.00)	1.04 (-1.13, 3.26)	0.86 (-1.36, 3.14)	2.62 (0.76, 4.52)
90th percentile	1.75 (0.32, 3.21)	0.72 (-1.26, 2.74)	0.05 (-5.05, 5.44)	-0.64 (-5.95, 4.97)	2.85 (-1.62, 7.52)
**Ca**					
10th percentile	1.75 (1.34, 2.16)	1.55 (0.83, 2.27)	1.50 (0.00, 3.02)	2.19 (0.50, 3.91)	2.75 (1.44, 4.07)
90th percentile	3.42 (2.08, 4.77)	2.15 (-0.39, 4.75)	1.62 (-3.60, 7.13)	3.92 (-2.04, 10.26)	4.12 (-0.52, 8.97)

Species	All causes	CVD	MI	Stroke	Respiratory diseases
**V**					
10th percentile	1.61 (1.22, 2.01)	1.56 (0.89, 2.23)	1.90 (0.51, 3.32)	1.82 (0.26, 3.40)	2.80 (1.60, 4.01)
90th percentile	1.92 (0.55, 3.32)	2.01 (-0.40, 4.47)	5.09 (0.08, 10.35)	0.32 (-5.35, 6.34)	4.94 (0.67, 9.38)
**Fe**					
10th percentile	1.79 (1.30, 2.28)	1.68 (0.85, 2.51)	1.68 (0.05, 3.34)	1.74 (-0.07, 3.57)	2.62 (1.19, 4.07)
90th percentile	2.65 (1.23, 4.09)	2.30 (-0.21, 4.86)	2.36 (-2.30, 7.24)	0.46 (-4.66, 5.85)	2.99 (-1.25, 7.40)
**Ni**					
10th percentile	1.49 (1.11, 1.88)	1.41 (0.77, 2.04)	1.25 (-0.09, 2.61)	1.70 (0.25, 3.17)	2.22 (1.08, 3.37)
90th percentile	1.57 (0.63, 2.51)	1.55 (-0.14, 3.27)	0.67 (-2.70, 4.16)	0.02 (-3.52, 3.70)	1.86 (-1.10, 4.91)
**Cu**					
10th percentile	1.56 (1.17, 1.96)	1.47 (0.82, 2.12)	1.71 (0.36, 3.09)	1.95 (0.52, 3.40)	2.52 (1.32, 3.74)
90th percentile	1.91 (0.89, 2.94)	1.99 (0.15, 3.86)	2.70 (-1.09, 6.64)	2.61 (-0.87, 6.20)	3.03 (-0.33, 6.50)
**Zn**					
10th percentile	1.49 (1.05, 1.92)	1.24 (0.55, 1.93)	1.12 (-0.29, 2.55)	1.90 (0.23, 3.59)	2.44 (1.10, 3.80)
90th percentile	1.82 (0.40, 3.27)	1.38 (-0.96, 3.78)	0.01 (-4.73, 4.99)	1.39 (-3.70, 6.75)	1.65 (-2.34, 5.81)

Figure S1. Estimated percent difference in mortality in association with for a 10-μg/m^3 increase in $PM_{2.5}$ at lag 0-1 by cause of death and season.

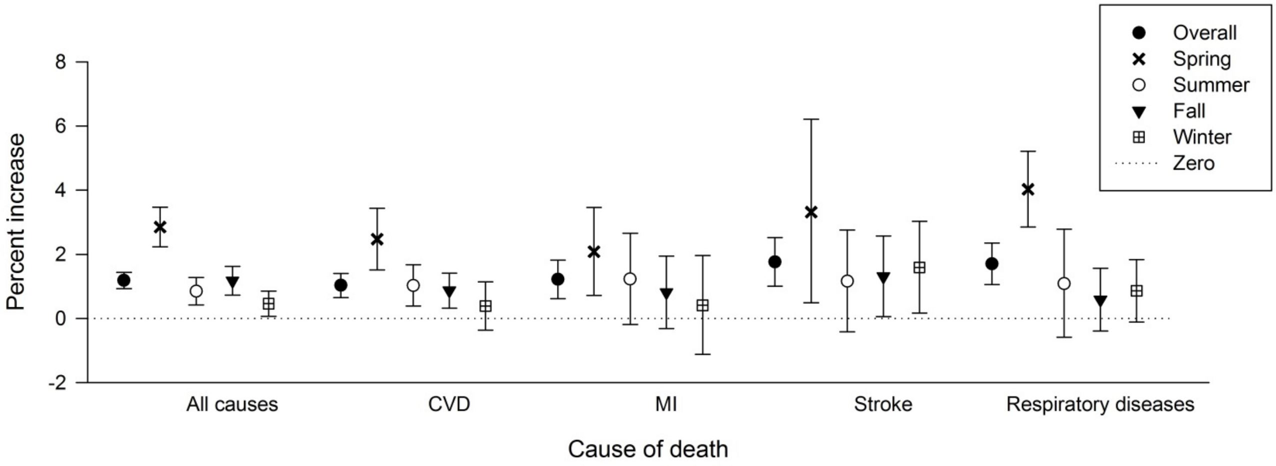

Figure S2. Distribution of county-level percentage of smoking and alcohol consumption.

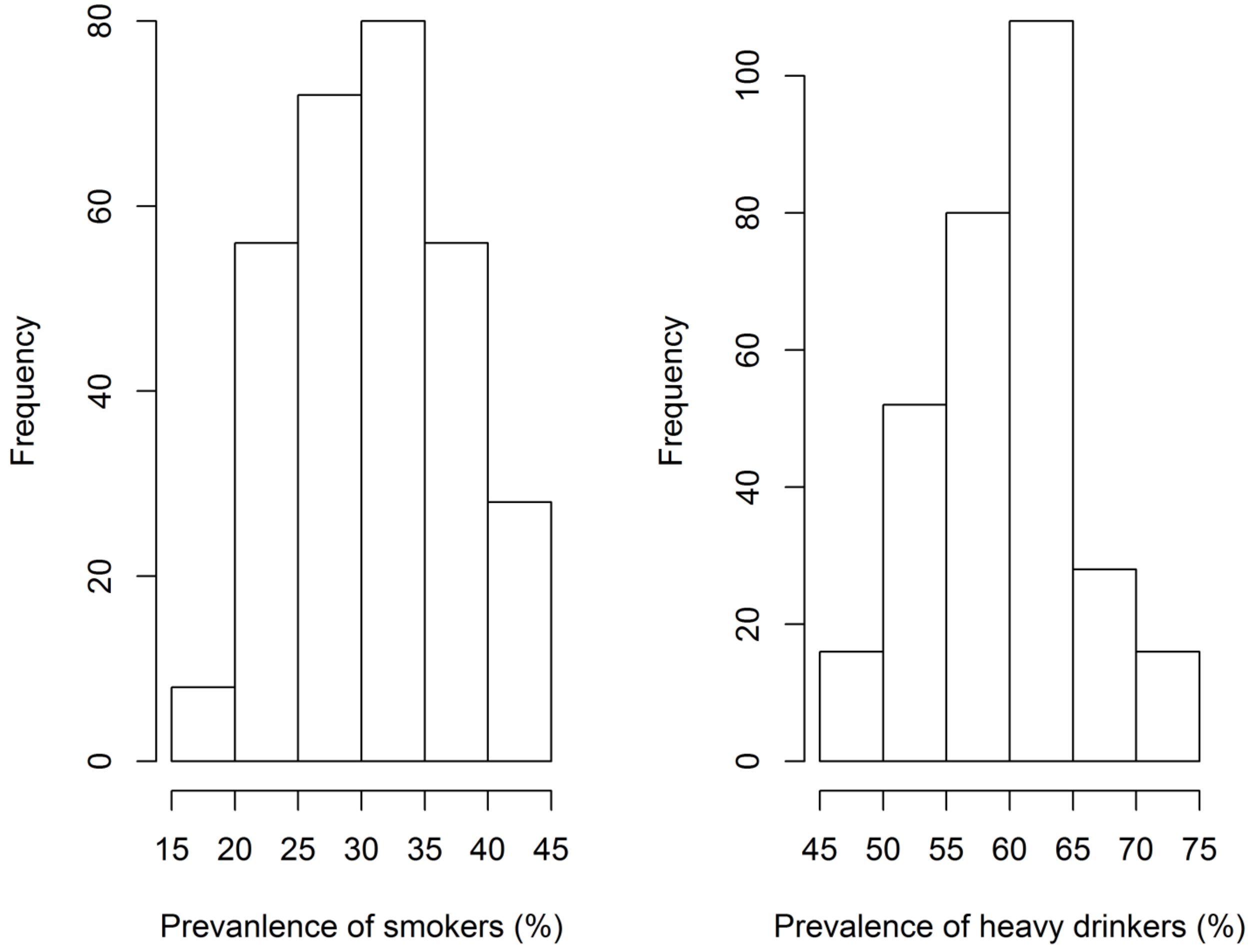

Supplement: (487 KB) PDF [file ehp.1307568.s001.pdf]
